# Individual and Environmental Factors Affecting Adaptive Behavior of Toddlers with Autism Spectrum Disorder: Role of Parents’ Socio-cultural Level

**DOI:** 10.1007/s10803-020-04803-x

**Published:** 2020-12-23

**Authors:** Giulia Balboni, Alice Bacherini, Gessica Rebecchini, Romina Cagiano, Alice Mancini, Raffaella Tancredi, Roberta Igliozzi, Filippo Muratori

**Affiliations:** 1 Department of Philosophy, Social and Human Sciences, and Education, University of Perugia, Piazza G. Ermini, 1, 06123 Perugia, Italy; 2 IRCCS Stella Maris Foundation, Via dei Giacinti, 2, Calambrone, 56018 Pisa, Italy; 3 Department of Clinical and Experimental Medicine, University of Pisa, Via dei Giacinti, 2, Calambrone, 56018 Pisa, Italy

**Keywords:** Toddler, Cultural capital, Social capital, Socio-Economic Status, Parent, Adaptive behavior

## Abstract

The effects of environmental factors [including Socio-Economic Status, Cultural Capital, and Social Capital (Socio-Cultural Level) of both parents] on the Vineland-II adaptive behavior dimensions of toddlers with autism spectrum disorder (ASD), in addition to individual factors, was investigated in 148 Italian toddlers (82% males), aged 18 to 37 months with ASD. Toddlers’ age and Griffiths Mental Development Scales general development affected all of the adaptive behavior dimensions, with negative and positive associations, respectively. The Child Behavior Checklist comorbid conditions were negatively associated with some adaptive behavior dimensions while the ADOS-2 Social affect only with the communication dimension. Mothers’ and fathers’ specific Socio-Cultural Level dimensions were positively associated with toddlers’ specific adaptive behavior dimensions with the same magnitude as comorbid conditions.

Autism spectrum disorder (ASD) is a neurodevelopmental disorder characterized by deficits in communication and social interaction and restricted and repetitive patterns of behavior, interests, or activities (American Psychiatric Association 2013). This disorder occurs in the early developmental period and can lead to impairments in adaptive behavior (e.g., Balboni et al. 2016b; Carter et al. 1998; Volkmar et al. 1993). Adaptive behavior is defined as “the collection of conceptual, social, and practical skills, that have been learned and are performed by people in everyday life” (Tassé et al. 2012, p. 291). Previous studies have reported that the Vineland Adaptive Behavior Scales-Second Edition (Vineland-II; Sparrow et al. 2005) with Communication, Daily living skills, Socialization, and Motor skills domains are valid for measuring the three conceptual, practical and social adaptive behavior domains, as well as motor skills, respectively (Sparrow et al. 2005). Adaptive behavior can be learned, and its expression increases the probability of social inclusion of the individual (Tassé and Balboni 2021) and the improvement of his/her quality of life (Balboni et al. 2020). Therefore, it is important to describe the factors that may influence the development and expression of adaptive behavior of individuals with ASD at an early stage of life. These factors are usually classified into individual and environmental factors which concurrently affect adaptive behavior. Several studies have investigated the relationship between individual factors (e.g., age, developmental level or autism symptom severity) and adaptive behaviors (e.g., Paul et al. 2014; Ray-Subramanian et al. 2011). However, research is lacking on the connection between environmental factors (e.g., socio-cultural background) and adaptive behavior as is research into the relationship of individual and environmental factors, when simultaneously considered, with adaptive behavior.

## Relationships Between Environmental Factors and Adaptive Behavior of Toddlers with ASD

The Socio-Cultural Level (SCL) is an environmental factor that requires further investigation. This factor is strictly related to the enduring reciprocal interactions between the individuals and their immediate environment, defined as proximal processes by Bronfenbrenner and Evans (2000). Based on Lamont and Lareau’s (1988) definition, SCL reflects the knowledge, preferences, behaviors, and goods characterizing the way of life of an individual or the adults forming a family, and depends on their cultural, social, and economic resources. SCL is a multidimensional construct composed of the three dimensions: Socio-Economic Status (SES), Cultural Capital, and Social Capital. The SES indicates the position of an individual within a social system in which social values as occupational prestige, educational level, and economic resources are not equally distributed (Bornstein and Bradley 2003). SES is generally evaluated through educational level, occupation (e.g., type, prestige or social status of the occupation), and income. Cultural Capital refers to the knowledge and use of cultural codes which are considered relevant in the community in which people live (Lamont and Lareau 1988). Consistent with the literature, Cultural Capital refers to three dimensions (Balboni et al. 2019): cultural activities (i.e., attending cultural events such as musical events and theater performances, visiting museums, reading books) and goods (books or artwork) (e.g., Dumais 2002); cultural technical skills and knowledge (i.e., using foreign languages, using the Internet to stay informed, performing in concerts, plays or dance productions, creating art) (Lareau and Weininger 2003); and participation in activities of cultural, community service, religious or political groups/associations (Jeannotte 2003). Social Capital refers to the resources associated with durable and trustworthy social network connections (Bourdieu 1986). It is composed of two dimensions: bonding Social Capital, which refers to the resources associated with the relationships of the individual with family members, relatives, friends, and colleagues, and bridging Social Capital, which refers to the resources associated with the relationships of the individual with groups or associations (i.e., economic, social, cultural, recreational, religious, political) in their own community.

Several previous studies have investigated the influence of maternal SES on cognitive development of toddlers with typical development. These studies found that a high educational level of the mother during the first years of life of their children is associated with better cognitive skills of the children (e.g., Arterberry et al. 2007; Koutra et al. 2012; Westerlund and Lagerberg 2008).

Regarding children with ASD, several studies examined the influence of SES on the risk of developing ASD (Bhasin and Schendel 2007; Segev et al. 2019; Wang et al. 2018), or associated comorbid conditions (Flouri et al. 2015; Dickerson Mayes and Calhoum 2011). One previous study examined the effects of mothers’ participation in cultural activities on the behavior of 4-to-6-year-old children with ASD, reporting that mothers’ participation in cultural activities was associated with better social and daily living skills of their offspring (Avrech Bar et al. 2016). No previous studies have examined the effects of any dimension of mothers’ and fathers’ SCL on the behavior of toddlers with ASD.

Regarding other environmental factors, previous investigations have explored the influence of parental socio-demographic characteristics, such as parents’ age, on the adaptive behavior of children with ASD (Vierck and Silverman 2015). Several previous studies reported no statistically significant relationships between both maternal and paternal age and the adaptive behavior of their offspring with ASD (Ben Itzchak et al. 2011; Lyall et al. 2020; Vierck and Silverman 2015).

## Relationships Between Individual Factors and Adaptive Behavior of Toddlers with ASD

Several individual factors, such as demographic characteristics (age, birth order) or clinical characteristics (developmental level, autism symptom severity, and associated comorbid conditions) have been found to have relationships with the adaptive behavior of toddlers with ASD (Paul et al. 2014; Ray-Subramanian et al. 2011).

Previous studies have consistently reported the presence of negative associations between children’s age and adaptive behavior in toddlers (Nevill et al. 2017; Uljarevic et al. 2018) as well as in preschoolers (Perry et al. 2009) and in older children with ASD (Kanne et al. 2011). In contrast, a previous study of the association between birth order and adaptive behavior reported no differences on the Vineland Adaptive Behavior Scale (Sparrow et al. 1984) between scores of first-born and second-born siblings with ASD (Berends et al. 2019).

Several studies have reported that developmental level is positively related to adaptive behavior in toddlers with ASD (Nevill et al. 2017; Ray-Subramanian et al. 2011; Uljarevic et al. 2018). In contrast, autism symptom severity has been reported to exhibit a negative relationship with adaptive behavior of toddlers with ASD (Paul et al. 2014; Ray-Subramanian et al. 2011; Uljarevic et al. 2018). Specifically, total score on the scale of autism symptom severity Autism Diagnostic Observation Schedule-Second Edition (ADOS-2; Lord et al. 2012) was found to be negatively related to the Communication and Daily living skills Vineland-II domains, but not to the Socialization and Motor skills domains in toddlers with ASD (Paul et al. 2014; Ray-Subramanian et al. 2011). However, other studies of toddlers with ASD found no relationships between ADOS-2 Social affect and the Restricted and repetitive behavior domains with the Vineland-II scales (Nevill et al. 2017).

A small number of studies have investigated the associations between comorbid conditions and adaptive functioning of toddlers or preschoolers with ASD, reporting that the presence of both internalizing (e.g., anxiety, depression, withdrawal, and psychosomatic complaints) and externalizing behaviors (e.g., aggression, attention problems, hyperactivity, and impulsiveness) is related to greater impairments of adaptive skills (Hartley et al. 2008; Uljarevic et al. 2018).

However, the relationships between individual and environmental factors and the adaptive behavior of children with ASD in the early stage of life are still unclear. The present study aimed to investigate whether there was any relationship between adaptive behavior of toddlers with ASD aged 18 to 37 months and individual factors (age, birth order, general development, autism symptom severity, and comorbid conditions) or environmental factors (SES, Cultural Capital, Social Capital, and age of mother and father), when considered simultaneously. To examine this issue, we first investigated the relationship between each individual and environmental factor with each adaptive behavior domain. We then studied which of the individual and environmental factors that had resulted to be significantly related to adaptive behavior affected each adaptive behavior dimension, when considered simultaneously. Individual and environmental factors do not act independently but simultaneously in affecting the expression and development of the adaptive behavior. Therefore, exploring these factors simultaneously is of paramount importance. The efficacy of interventions based only on individual factors could be limited by the effects of environmental factors. Knowing as these elements interact may be useful to have a complete picture of the factors that are at increased risk for weaknesses in adaptive behavior and that therefore must be taken into account when planning early interventions for toddlers with ASD.

## Methods

### Materials

#### Adaptive Behavior

Adaptive behavior was measured using the Vineland-II Survey Interview Form (Sparrow et al. 2005) which allows the assessment of adaptive behavior of individuals between the ages of 0 and 90 years. Each of the four domains comprise multiple subdomains: Communication (Receptive, Expressive and Written subdomains), Daily living skills (Personal, Domestic and Community subdomains), Socialization (Interpersonal relationships, Play and leisure time and Coping skills subdomains), and Motor skills (Gross and Fine subdomains). A Vineland-II Composite scale is available, providing an assessment of the overall adaptive behavior level. Standard Scores (*M* = 100; *SD* = 15) are available for domains and Composite Scale and *v*-scale scores (*M* = 15; *SD* = 3) for subdomains. In the current study, we used an Italian adaptation of the Vineland-II, approved by the Pearson Editor, with excellent psychometric properties and normative scores published in 2016 (Balboni et al. 2016a).

#### General Development

General development of children at this early age was assessed using the Griffiths Mental Development Scales (GMDS). Two versions of the GMDS revised edition are available: Griffiths Mental Development Scales Revised (GMDS-R; Griffiths 1996; Italian adaptation, Battaglia and Savoini 2007) and Griffiths Mental Development Scales Extended Revised (GMDS-ER; Luiz et al. 2006; Italian adaptation, Cianchetti and Sannio Fancello 2007) for children aged 0 to 24 months or 24 to 36 months, respectively. Both the GMDS-R and GMDS-ER are composed of five scales: Locomotor (which measures gross motor skills), Personal-social (which measures autonomy, daily living skills, and social interaction), Hearing and language (which measures both receptive and expressive language), Eye and hand co-ordination (which measures fine motor and visual-spatial skills), and Performance (which measures the ability of manipulate objects). A general development index (General Quotient, GQ) is available (*M* = 100; *SD* = 15).

#### Autism Symptoms

The ADOS-2 (Lord et al. 2012; Italian adaptation, Colombi et al. 2013) was used to assess symptoms of autism through a standard series of activities designed to elicit certain behaviors. The ADOS-2 includes five modules that are used based on the child’s expressive language level and chronological age. For individuals 18 to 37 months of age, the following Modules are available: Toddler module for individuals aged 12 to 30 months; Modules 1, 2 and 3, for individuals older than 30 months who do not speak with phrases, those who speak with phrases but not fluently, and those who speak fluently, respectively. Calibrated Severity Scores (range: 0–10) are available for the two domains Social affect and Restricted and repetitive behavior, which together produce the Total score (Esler et al. 2015; Hus et al. 2014). Scores between 8 and 10 indicate a high level of autistic symptoms, scores between 5 and 7 indicate a mild level of symptoms, scores between 3 and 4 indicate a low level of symptoms, and scores between 1 and 2 indicate a minimum level or absence of symptoms. Calibrated Severity Scores have been developed to be used instead of ADOS total scores (i.e., the sum of raw scores on diagnostic items) as a standardized measure of ASD symptoms, because Calibrated Severity scores are influenced less than total scores by the individual’s age, verbal and non-verbal development, and mother’s educational level (de Bildt et al. 2011; Gotham et al. 2009; Hus Bal and Lord 2015; Shumway et al. 2012). Calibrated Severity Scores are therefore used to compare assessment across ADOS modules. Recently, it was found that for pre-school children ADOS Calibrated Severity Scores are the best measure of ASD symptoms and are less affected by co-occurring conditions and demographic features (Wiggins et al. 2019). Therefore, caution in using them for non-verbal young children expressed Hedley et al. (2016), now appears unwarranted.

#### Comorbid Conditions

The parent report Child Behavior Checklist 1½-5 (CBCL 1½-5; Achenbach and Rescorla 2000; Italian adaptation, Frigerio et al. 2006) was used to evaluate comorbid conditions of toddlers (Muratori et al. 2019). The item scores are aggregated in (a) three summary scales: Internalizing, Externalizing, and Total problems; (b) seven syndrome scales: Emotionally reactive, Anxious/Depressed, Somatic complaints, Withdrawn, Sleep problems, Attention problems, and Aggressive behavior; and (c) five DSM-oriented scales consistent with diagnostic categories of the DSM-IV and DSM-5: Affective problems, Anxiety problems, Pervasive developmental problems, Attention deficit/Hyperactivity problems, and Oppositional defiant problems. For the summary scales, T scores of 63 and above are considered clinically significant; values between 60 and 63 indicate the borderline range; values under 60 are considered non-clinical.

#### Socio-Economic Status

SES was measured through years of education completed and occupational prestige. Occupational prestige was assessed with the Italian Occupational Prestige Scale (Meraviglia and Accornero 2008), an ordinal scale that allows the classification of occupations in 110 occupational categories ordered according to the prestige associated with each occupation as a score ranging from 10.84 to 89.93.

#### Cultural Capital

The Scale of Cultural Capital was used (Balboni and Cubelli 2016; Balboni et al. 2019). This scale is a self-report questionnaire composed of 14 items with a 5-point Likert scale (0 to 4). The Scale of Cultural Capital was developed in accord with the literature for the measurement of the three main dimensions of Cultural Capital (Balboni et al. 2019): (a) participating, which refers to the participation and membership in social, political, religious and cultural groups/associations (four items); (b) consuming, which refers to cultural activities, such as visiting museums, exhibitions and art galleries, attending theater performances and musical events, and having books and reading for pleasure (five items); (c) expert using, which refers to cultural activities that require technical skills and formal experience, like reading books for study or work, attending courses and seminars, using foreign languages and using the Internet to stay informed, writing and producing artwork or performing in concerts, plays or dance productions (five items). The total score ranges from 0 to 56. The factor structure of the Scale of Cultural Capital was verified using exploratory and confirmatory factor analyses and its invariance across sex and occupational prestige was verified via multigroup confirmatory factor analyses. The three factors showed good reliability and convergent/divergent validity when compared with the bonding and bridging Social Capital dimensions (Balboni et al. 2019).

#### Social Capital

To assess Social Capital, we used the Personal Social Capital Scale (Chen et al. 2009; Italian adaptation, Balboni et al. 2011). This scale is a self-report questionnaire composed of 10 items (divided into 54 sub-items) using a 5-point Likert scale that allows for the measurement of bonding and bridging Social Capital (five items each). The total score ranges from 54 to 270. Chinese and English versions of this scale exhibit excellent reliability and construct validity (Chen et al. 2009). For the Italian version, the items also exhibit good psychometric properties (see Menardo et al. (2017) for an example of the use of the Italian version).

#### Social Desirability

The short form of the Balanced Inventory of Desirable Responding-Italian version (BIRD-6; Bobbio and Manganelli 2011; Paulhus 1991) was used to detect attempts at simulation. This measure is made up of 16 items with a 6-point Likert scale to evaluate the unconscious tendency to provide honest but positively-biased responses, as well as the habitual and conscious presentation of a favorable public image. Individuals with a total score exceeding the 95th centile of the normative sample were identified as simulators. This scale is reported to show adequate reliability and validity (Bobbio and Manganelli 2011).

The psychometric properties of the Scale of Cultural Capital, Personal Social Capital Scale and BIRD-6 were verified with Italian adults 18 to 70+ years old, approximately half males and half females, with different educational levels and occupational status (Balboni et al. 2019; Bobbio and Manganelli 2011; Menardo et al. 2020). All of these instruments have also been used in a similar study on the effects of parents’ SCL on personality profile of offspring (Menardo et al. 2017).

### Participants

Participants were 148 Italian children (82% males) aged 18 to 37 months old (*M* = 30.84, *SD* = 4.62) with a diagnosis of ASD. Seventy-four participants (50%) were only children. Of the remaining participants, 22 (15%) were first-born, 46 (31%) were second-born, four (3%) were third-born, and two (1%) were fourth-born children. Participants were recruited between February 2016 and April 2019 among children who had been evaluated for suspected ASD at the Autism Spectrum Disorders Unit of the IRCCS Stella Maris Foundation, an Italian Children’s Neuropsychiatric Hospital. Toddlers were selected using the following inclusion criteria: (a) receiving a diagnosis of ASD during a clinical evaluation process at the IRCCS Stella Maris Foundation; (b) having no associated major physical disorders (e.g., visual, motor or hearing impairment, epilepsy), no known genetic syndromes (e.g. Down, X-fragile Syndrome), and not being born prematurely (i.e., before 32 weeks of pregnancy); (c) being born in Italy and having at least one Italian parent; (d) not having siblings already involved in the present study; (e) being evaluated for adaptive behavior and for each individual and environmental factor in the present study during their stay at IRCCS Stella Maris Foundation.

Originally, 181 parents of toddlers agreed to being involved in the present study. However, 33 toddlers were excluded because at the end of the evaluation process they were classified as having a non-ASD neurodevelopmental disorder (*n* = 11) or an associated major physical disorder (*n* = 2), or were not assessed for at least one individual factor (*n* = 20; for 19 toddlers, it was not possible to administer the ADOS-2 or GMDS, or both; for one toddler’s parent, the CBCL1½-5 was not administered).

As shown in Table 1, 148 toddlers had a medium level of autistic symptomatology (as measured by the ADOS-2 Total scale), a general development level (as measured by the Griffiths Mental Development Scales) below the average of the normative group (a normative score was available for only 27% of participants, and they had a General Quotient *M[SD]* = 79.34 [14.46]; for the other 73%, the obtained raw score was below the minimum raw score for which a normative score is available), a non-clinical level of behavioral and emotional problems (as measured by the CBCL1½-5 Total problems scale), and a level of adaptive behavior (as measured by the Vineland-II Composite scale) more than 1 standard deviation lower than that of the general population.

**Table 1:** Characteristics of toddlers with ASD (*n* = 148)

Measure	Mean (*SD*)
ADOS-2 (Calibrated Severity Score)	
Social affect	6.68 (2.01)
Restricted and repetitive behaviors	7.53 (2.24)
Total	7.00 (2.15)
Griffiths Mental Development Scale (raw score^a^)	
Total	240.34 (35.38)
CBCL 1½-5 (T-score)	
Total problems scale	58.19 (10.32)
Vineland-II scales (Standard Score)	
Communication	58.05 (13.88)
Daily living skills	73.53 (11.65)
Socialization	73.03 (10.90)
Motor skills	90.08 (13.21)
Composite scale	74.42 (10.75)

^a^A normative score for the Griffiths Mental Development Scale was only available for 27% of participants, and they had a General Quotient *M(SD)* = 79.34(14.46); for the other 73%, the obtained raw score was below the minimum raw score for which a normative score is available

Of the 148 toddlers, 102 (69%) had at least one current intervention: 71 toddlers had only one current intervention, while the remaining had two (25 toddlers), three (5 toddlers) or 4 (just one toddler) current interventions. These interventions were psychomotricity (51%), speech therapy (19%), Applied Behavior Analysis (13%), Early Start Denver Model (7%), multisystemic water therapy (5%), other intervention (DIR, Thérapie d'échange et de développement/TED or educational) (4%), and music therapy (1%). The majority of these interventions (69%) were started within the previous 1–6 months.

For each of the 148 toddlers, both parents (except one father for a single mother family) were assessed for SCL. Investigating social desirability in parents, as measured by the BIDR-6, we identified and consequently excluded 14 mothers and 12 fathers for whom scores exceeded the cut-off for simulation. Table 2 shows the data for the remaining 134 mothers and the 135 fathers, including age, SES, investigated with educational level and occupational prestige measured with the Italian Occupational Prestige Scale, Cultural Capital and Social Capital measured with the Scale of Cultural Capital and the Personal Social Capital Scale, respectively.

**Table 2:** Characteristics of mothers (*n* = 134) and fathers (*n* = 135) of toddlers with ASD

Measure	Mothers	Fathers
Age		
Mean (*SD*)	35.84 (5.09)	39.53 (6.25)
Range	24–48	24–59
Socio-Economic Status		
Years of study		
Mean (*SD*)	14.20 (3.42)	13.29 (3.45)
Range	8–23	5–21
Occupational prestige (scale score range: 10.84–89.93)		
Mean (*SD*)	37.16 (20.85)	42.27 (20.14)
Range	10.84–89.66	10.84–89.66
Cultural Capital factors		
Participating (factor score range: 0–16)		
Mean (*SD*)	1.39 (2.20)	1.61 (2.41)
Range	0–10	0–12
Consuming (factor score range: 0–20)		
Mean (*SD*)	3.83 (2.35)	4.10 (3.11)
Range	0–10	0–18
Expert using (factor score range: 0–20)		
Mean (*SD*)	5.86 (2.85)	6.36 (3.16)
Range	1–16	1–16
Social Capital factors		
Bonding (factor score range: 0–150)		
Mean (*SD*)	89.01 (13.07)	94.63 (14.41)
Range	44–117	56–125
Bridging (factor score range: 0–120)		
Mean (*SD*)	54.59 (13.65)	55.97 (14.35)
Range	26–91	24–89

Of the 148 mothers and 147 fathers of the toddlers with ASD, 14 mothers and 12 fathers (one father did not belong in the child’s household) were excluded because their scores on the Balanced Inventory of Desirable Responding-6 Short Form exceeded the cut-off for simulation

Both parents had a mean number of years of study close to the high school diploma and an occupational prestige level in the middle range. Regarding Cultural Capital and Social Capital factors, we compared the scores of the mothers and fathers with those of 150 Italian mothers and 125 Italian fathers obtained using the same questionnaires (see Menardo et al. 2017). For the Scale of Cultural Capital, the mean scores obtained by the mothers and fathers in the present investigation were within 1 point from the mean of the reference group except for the Consuming factor, for which the mothers had a lower score, and for the Expert using factor, for which the fathers had a higher score. For the Personal Social Capital Scale, the mean scores of both parents were similar to those of the reference group for the bridging factor, but were more than 10 points lower for the bonding factor.

All parents provided written informed consent and their anonymity was guaranteed. Toddlers and parents did not receive any form of incentive to participate in this study.

### Procedure

Trained psychologists administered the GMDS-R (12%) or the GMDS-ER (88%) and the ADOS-2 Toddler Module (37%) or Module 1 (63%) in a counterbalanced order (GMDS-ADOS-2 in 51% of cases, ADOS-2-GMDS in 49% of cases). Graduate students with a major in clinical psychology or neurodevelopmental pediatricians administered the Vineland-II, interviewing the mother (88%) or the father (12%) of the toddlers. The CBCL 1½-5 was filled out by the same parent that was interviewed for the Vineland-II. The diagnosis of ASD or other neurodevelopmental disorders was conducted by a multi-interdisciplinary team in accord with the DSM-5 criteria and on the bases of the ADOS-2 scale score, direct observation, and parent interviews.

Both parents were interviewed regarding their years of education and type of occupation. Moreover, both parents filled out the Scale of Cultural Capital, the Personal Social Capital Scale, and the BIRD-6 scale of social desirability in a counterbalanced order, with the Social Desirability Scale always placed at the end (Cultural Capital-Social Capital in 47% of cases, Social Capital-Cultural Capital in 53% of cases).

### Data Analysis

In accord with Tabachnick and Fidell’s suggestions (Tabachnick and Fidell 2013), we used the linear interpolation technique to estimate any missing data in the measurement of parents’ SCL: 0.94% of item scores of the Scale of Cultural Capital and 0.04% of item scores of the BIDR-6 of mothers; 0.68% of occupational prestige scores, 0.05% of item scores of the Scale of Cultural Capital, 0.04% of item scores of the Personal Social Capital Scale, and 0.04% of item scores of the BIDR-6 of fathers.

Pearson’s correlation coefficients were calculated to investigate the relationships among each individual and environmental factor and each Vineland II domain and the Composite Scale. Children’s age and birth order, GMDS raw score, ADOS-2 Social affect and Restrictive and repetitive behavior Calibrated Severity Score, CBCL 1½-5 Total problems T-score were considered as individual factors; mothers’ and fathers’ age, years of education, occupational prestige scores, Scale of Cultural Capital participating, consuming, and expert using dimension scores and Personal Social Capital Scale bonding and bridging dimension scores were considered as environmental factors. In accord with Cicchetti et al. (2011), the magnitude of the correlation coefficients was evaluated as small (0.10–0.29), medium (0.30–0.49), large (0.50–0.69), or very large (≥ 0.70).

To investigate which individual and environmental factors affected the toddlers’ adaptive behavior when considered simultaneously, we ran two multiple regression analyses for each of four Vineland-II domains and for the Composite scale. Each Vineland-II domain or the Composite scale was the dependent variable. All of the individual and maternal (first regression) or paternal (second regression) environmental factors that were found have a statistically or tendentially significant relationship with the Vineland-II domain or the Composite scale (*p* ≤  .08) were entered simultaneously as independent variables. The squared semipartial correlation coefficient (*sr*^*2*^) of each statistically significant independent variable was computed to detect its unique contribution to the total explained variance of toddlers’ adaptive behavior. Benjamini and Hochberg’s correction for multiple comparisons (Benjamini and Hochberg, 1995) was applied; however, the appropriate level of significance remained at *p* < .05.

Following Tabachnick and Fidell’s suggestions, regression assumptions were ascertained previously running the regression analyses in accordance with a five-step procedure. (1) Appropriateness of the participants size was investigated in accordance with the assumption that *N ≥ *104 + *m* (where *m* was the number of independent variables); (2) presence of univariate outliers (e.g., participants with *z* values higher than |3.29|) and multivariate outliers (e.g., participants for which the probability associated with the Mahalanobis distance was lower than .001) was checked for all the independent and dependent variables. Normality of the univariate distribution was verified by computing asymmetry and kurtosis values, considering as appropriates indices included in the range of − 1.00 to 1.00. Normality of the multivariate distribution was verified using Mardia’s test; (3) multicollinearity among independent variables was investigated by computing the tolerance index and the variance inflation factor (VIF), and the absence of collinearity was considered for values higher than .50 and lower than 2, respectively; (4) normality, linearity and homoscedasticity of errors were verified. We examined the shape of the residual distribution scatterplots, comparing the scatterplots of the obtained residual values with those of the theoretical values provided by Tabachnich and Fidell (2013), both for the set of independent variables and for each dependent variable; (5) independence of errors was investigated via Durbin–Watson statistics, considering appropriate values included in the range of 1.5–2.2, and the presence of outliers (e.g., extreme values higher than |3.29|) was detected via the analysis of the standardized residuals.

Normalized scores for the measurement of individual and environmental factors were used in all analyses.

## Results

### Relationships Between Individual and Environmental Factors with Adaptive Behavior Domains of Toddlers with ASD

As shown in Table 3, among the individual factors, toddlers’ general development level (GMDS) was the only factor that was positively statistically significantly related to all of the adaptive behavior dimensions and the Composite scale with a medium or small (only for Motor skills) correlation coefficient magnitude. Both toddlers’ age and comorbid conditions (CBCL 1½-5) were statistically significantly negatively related to all of the Vineland-II domains and the Composite scale with correlation coefficients of medium magnitude (except for the correlations for Daily living skills/age and Motor skills/comorbid conditions, for which the magnitude was small). The ADOS-2 Social affect dimension had statistically significant negative correlations (with coefficients of medium magnitude) with all of the dimensions of adaptive behavior, except for the Motor skill domain. Finally, toddlers’ birth order and ADOS-2 Restrictive and repetitive behavior exhibited no significant relationships with any adaptive behavior domain.

**Table 3:** Pearson’s correlation coefficients for toddlers’ with ASD individual and environmental factors and Vineland-II domains and Composite scale

		Vineland-II									
		Communication		Daily living skills		Socialization		Motor skills		Composite scale	
Individual factors											
Toddlers’ (*n* = 148)											
Age		− .33**		− .27**		− .32**		− .39**		− .37**	
Birth order		− .10		− .08		− .11		− .02		− .07	
General development											
GMDS total		.44**		.50**		.35**		.29**		.47**	
Autism symptoms											
ADOS-2 Social affect		− .40**		− .37**		− .33**		− .14		− .36**	
ADOS-2 Restricted and repetitive behaviors		− .08		− .09		− .06		− .09		− .10	
Comorbid conditions											
CBCL 1½-5 Total problems		− .36**		− .35**		− .42**		− .17*		− .37**	

Environmental factors		Mothers	Fathers	Mothers	Fathers	Mothers	Fathers	Mothers	Fathers	Mothers	Fathers
Mothers’ (*n* = 134) or fathers’ *(n* = 135)											
Age		− .05	.07	.03	.08	− .04	.04	− .10	− .03	− .05	.04
Socio-Economic Status											
Years of study		.26**	.08	.16°°°	.07	.22*	.11	.11	− .10	.22*	.05
Occupational prestige		.12	.22*	.15	.13°	.09	.19*	.11	.02	.14°°°	.16°°°
Cultural Capital factors											
Participating		.03	− .03	.11	− .03	.02	− .08	.06	− .10	.06	− .06
Consuming		.11	.03	.16°°°	.12°	.13	.05	.14	.01	.16°°	.07
Expert using		.07	.13	.11	.12	.14°°°	.20*	.05	.00	.10	.14
Social Capital factors											
Bonding		.01	.02	.12	.10	.08	.03	− .02	− .09	.06	.02
Bridging		.09	− .13°	.20*	.03	.15°	− .09	.07	− .09	.14°°°	− .07

**p ≤ .01; *p ≤ .05; °°°*p* ≤ .06; °°*p* ≤ .07; °*p* ≤ .08

Regarding environmental factors, mothers’ years of study were statistically significantly related to Communication and Socialization domains and the Composite scale. Fathers’ occupational prestige was statistically significantly associated with the Vineland-II Communication and Socialization domains. Fathers’ expert using dimension of Cultural Capital was statistically significantly related to the Vineland-II Socialization domain, whereas mothers’ bridging dimension of Social Capital was significantly related to the Vineland-II Daily living skills domain. All of these statistically significant correlation coefficients were positive, and had a small magnitude.

### Individual and Environmental Factors Affecting Adaptive Behaviors of Toddlers with ASD

The factors that were statistically or tendentially significantly correlated with the toddler’s adaptive behavior were: four individual factors (toddlers’ age, general development level, social affect autism symptoms, and total comorbid conditions), five maternal and four paternal environmental factors (occupational prestige, consuming and expert using Cultural Capital dimensions, and bridging Social Capital dimension of both parents, and mothers’ years of study). Therefore, maximum nine independent variables were introduced in the regression models for the individual and maternal factors regression analyses, and maximum eight independent variables were introduced for the individual and paternal factors regression analyses. Accordingly, the required minimum number of 113 and of 112 participants for the individual-maternal and the individual-paternal factors regression analyses was satisfied. Neither univariate nor multivariate outliers were found, and the normality of the univariate and multivariate distribution of the scores on the measurement of the individual and environmental factors were satisfied. Among all the independent variables, the absence of multicollinearity was ascertained because all of the tolerance and VIF index values were higher than .50 and lower than 2, respectively. For each independent variable taken separately as well as within the set of predictors, normality, linearity and homoscedasticity of the residuals was ascertained. The Durbin–Watson values exhibited no autocorrelations among the residuals. Finally, no outliers in the standardized residuals were detected.

Table 4 shows the results of the multiple regression analyses run to investigate individual and maternal or paternal environmental factors affecting each adaptive behavior domain when considered simultaneously. For each Vineland-II domain and Composite scale, two regression analyses were run. Independent variables were all of the individual factors and the maternal (for the first regression) or paternal (for the second regression) environmental factors which resulted in a statistically or a tendentially significant relationship with the adaptive behavior domain being considered. The dependent variable was each adaptive behavior domain and Composite scale.

**Table 4:** Standard multiple regressions of individual and environmental factors on Vineland-II domains and Composite scale of toddlers with ASD

	Vineland-II									
	Communication		Daily living skills		Socialization		Motor skills		Composite scale	
	*β*	*sr*^*2*^	*β*	*sr*^*2*^	*β*	*sr*^*2*^	*β*	*sr*^*2*^	*β*	*sr*^*2*^
Individual and maternal environmental factors (*n* = 134)										
Toddlers’										
Age	− .52***	.23	− .47***	.19	− .48***	.20	− .55***	.27	− .58***	.29
General development										
GMDS total	.47***	.13	.56***	.17	.40***	.09	.46***	.18	.58***	.19
Autism symptoms										
ADOS-2 Social affect	− .17*	.02	− .08		− .10				− .05	
Comorbid conditions										
CBCL 1½-5 Total problems	− .16*	.02	− .16*	.02	− .26***	.06	− .06		− .17**	.03
Mothers’										
Socio-Economic Status										
Years of study	.16*	.02	.00		.08				.08	
Occupational prestige									.01	
Cultural Capital factors										
Consuming			.07						.07	
Expert using					.04					
Social Capital factors										
Bridging			.15*	.02	.08				.06	
Adjusted *R*^*2*^	.55		.51		.47		.36		.61	
*F*	33.35***		20.93***		18.12***		26.12***		26.85***	
Individual and paternal environmental factors (*n* = 135)										
Toddlers’										
Age	− .53***	.23	− .48***	.19	− .46***	.17	− .55***	.29	− .57***	.28
General development										
GMDS total	.46***	.12	.58***	.19	.41***	.09	.47***	.19	.59***	.20
Autism symptoms										
ADOS-2 Social affect	− .19*	.02	− .05		− .05				− .04	
Comorbid conditions										
CBCL 1½-5 Total problems	− .14*	.02	− .16*	.02	− .26***	.06	− .01		− .17**	.02
Fathers’										
Socio-Economic Status										
Occupational prestige	.09		− .06		− .02				.02	
Cultural Capital factors										
Consuming			.14*	.02						
Expert using					.12					
Social Capital factors										
Bridging	− .08									
Adjusted *R*^*2*^	.54		.47		.41		.35		.56	
*F*	27.32***		20.85***		16.77***		24.76***		34.60***	

***p ≤ .001; **p ≤ .01; *p ≤ .05

The results revealed that toddlers’ age and comorbid conditions were found to negatively affect all of the Vineland-II domains and the Composite scale, except comorbid conditions, which did not affect the Motor skill domain. In contrast, toddlers’ general development positively affected all of the adaptive behavior domains and the Composite scale. Interestingly, ADOS-2 Social affect, although it was significantly correlated with all of the Vineland-II domains (except Motor skills), did not affect any adaptive behavior domains when considered simultaneously with the other individual and environmental factors (except for a negative effect on the Communication domain).

Regarding environmental factors, we found that when entered along with individual factors in the regression model, mothers’ years of study and bridging Social Capital positively affected toddlers’ Communication and Daily living skills domains, respectively. Fathers’ consuming Cultural Capital factor positively affected toddlers’ Daily living skills domain.

Overall, as indicated by the squared semipartial correlation coefficients (*sr*^*2*^), among both individual and environmental factors, toddlers’ age and general development level appeared to be the independent variables that most strongly affected all, or almost all, dimensions of children’s adaptive behavior, with squared semipartial correlation coefficients ranging from .17 to .29 and from .09 to .20, respectively. Toddlers’ comorbid conditions and parents’ socio-cultural dimensions made a contribution to specific toddlers’ adaptive behavior domains, with squared semipartial correlation coefficients ranging from .02 to .06 and equal to .02, respectively.

## Discussion

The current study was conducted to investigate the specific roles of individual factors (age, birth order, developmental level, autism symptom severity, comorbid conditions) and environmental factors (SES, Cultural Capital, Social Capital and age of both mothers and fathers), considered simultaneously, in affecting the adaptive behavior of toddlers with ASD. Specifically, we sought to identify whether environmental factors as parents’ SCL dimensions were associated with the adaptive behavior dimensions of children with ASD at this early age.

Regarding the SCL of both parents, when considered simultaneously with the other factors, we found that mothers’ years of study positively affected the Vineland-II Communication domain, while both mothers’ bridging Social Capital and fathers’ consuming Cultural Capital factors positively affected the Daily living skills domain. Already at this early stage of life, the development of toddler’s communication skills was associated with the mother’s educational level. Similarly, the engagement of the child in daily activities was associated with greater involvement and connections of the mother with associations in her community and with the involvement of the fathers in cultural activities such as visiting museums, exhibitions and art galleries, attending theater performances and musical events, and having books and reading for pleasure. It is possible that involvement in cultural activities and associations provide the parents with opportunities to discuss and understand other parents’ experiences, or being less strictly focused on their children’s difficulties. Thus, socially engaged parents might allow their children a higher level of independence and more chances to experiment with daily living skills.

No statistically significant association was found among both parents’ chronological age and toddlers’ adaptive behavior. Previous studies found that advanced parental age is associated with an increased risk of ASD in the offspring (Wu et al. 2017). Our study indicates that advanced parental age is not an environmental risk factor for weakened adaptive behavior in toddlers with ASD, consistent with previous studies (Ben Itzchak et al. 2011; Lyall et al. 2020; Vierck and Silverman 2015).

Among the toddlers’ individual factors, we found that, when considered with the environmental factors, toddler’s age and general development affected all of their adaptive behavior dimensions, with negative and positive relationships, respectively, in accord with other investigations with toddlers and older children with ASD (e.g., Perry et al. 2009; Ray-Subramanian et al. 2011; Uljarevic et al. 2018). Also toddlers’ comorbid conditions resulted in negative relationships with all of the adaptive behavior domains (but not with Motor skills), in accord with a previous study of older children (Hartley et al. 2008). Finally, socio-affective symptomatology was negatively associated only with the toddlers’ Vineland-II Communication domain, while the repetitive behaviors symptomatology was not associated with any adaptive behavior domains, in accord with previous studies (Balboni et al. 2016b; Nevill et al. 2017; Paul et al. 2014).

Interestingly, the unique contribution of the environmental factor years of study, bridging Social Capital and consuming Cultural Capital to explaining the total variance of toddlers’ adaptive behavior was smaller than that of the toddlers’ age and general development, but similar to that of comorbid conditions. Thus, also at this early stage of life, environmental factors such as mothers’ years of study and dimensions of parents’ SCL as mothers’ bridging Social Capital and fathers’ consuming Cultural Capital factors, are as relevant as individual factors in influencing the adaptive behavior of toddlers with ASD.

The present findings make a unique contribution with potentially important implications: not only toddlers’ age, general development, and comorbid conditions but also dimensions of the parents’ SCL should be taken into account when planning early interventions for toddlers with ASD. The efficacy of interventions based only on toddlers’ age, general development, and comorbid conditions may be limited by the effects of the parents’ SCL. Promoting parents’ Cultural Capital and Social Capital might encourage them to be involved in cultural activities and attend cultural events and connections with associations/groups in their own communities. Psychologists and neurodevelopmental pediatricians should be aware of the necessity of spending time with toddlers’ parents to support these aims: investigate the parental cultural capital and social capital, learn of the connected activities offered by their community, and ascertain parents’ interest and willingness to commit to such activities. Family involvement in cultural/community activities could represent a “low-cost intervention” to suggest to families along with evidence-based treatments to improve family functioning and possibly the effectiveness of treatment.

Future investigations should evaluate the relevance of the current findings for the development of toddlers’ adaptive behavior by investigating Cultural Capital factors and Social Capital factors in relation to activities that primarily occur online (e.g., courses or conferences attended online, cultural associations conducting online activities, such as Facebook/online discussion groups). Moreover, regarding the measurement of the general development with the GMDS, we used raw scores rather than normative scores, given that almost 75% of the participants obtained a score below the minimum raw score for which a normative score was available. Normative scores are based on age norms comparing the participant's performance to children of the same age, whereas raw scores would not take the participant’s age into account. Raw scores reflect both ability and age. However, the participant’s age was entered as an independent variable with the general development in the regression analyses. Therefore, the effect of age seems to have been properly taken into account in the regression models. Another limitation of the present study that should be taken into account when interpreting the current results concerns the absence of control groups of toddlers with typical development or other neurodevelopmental disorders. Future investigations should be conducted to describe the unique characteristics of ASD. It is clinically important to know if the relationships found in this study are unique to the ASD population or are more general to youth with atypical development. Furthermore, the relationship between dimensions of the parents’ SCL, toddlers’ adaptive behavior, health professional policies and institutional practices and intervention choices for toddlers with ASD merits further investigation. Finally, longitudinal studies may be useful for determining the effects of individual and environmental factors, particularly Cultural Capital, Social Capital and SES of mothers and fathers in the trajectories of the development of adaptive behavior of toddlers with ASD.

However, despite these limitations, the current study highlights the relevance of evaluating environmental factors in addition to individual factors to provide a complete picture of the limits and the resources to take into account in the planning of early interventions for toddlers with ASD. Thus, consideration of a toddler’s environment can increase the likelihood of developing interventions that increase the toddler’s quality of life and that of their family.
